# Effects of work-related digital technology on occupational health in the public sector: A scoping review

**DOI:** 10.1177/10519815251320274

**Published:** 2025-03-18

**Authors:** Carin Håkansta, Annica Asp, Kristina Palm

**Affiliations:** 1 Unit of Occupational Medicine, Karolinska Institutet, Stockholm, Sweden; 2 Department of Working Life Science, Karlstad university, Karlstad, Sweden

**Keywords:** digitalisation, occupational health, working conditions, employees, government, psychological well-being, social isolation, work-life balance

## Abstract

**Background:**

Despite a growing literature on how digitalisation affects service quality, justice, and accountability in the public sector, research on the effects on the work and work environment of public employees is scarce.

**Objective:**

To present and discuss existing evidence and identify knowledge gaps related to how digitalization affects the work and work environment of public sector employees.

**Methods:**

This scoping review is based on peer reviewed academic journal articles in English found in PubMed, PsycINFO, Business Source Premier (EBSCO) and Scopus.

**Results:**

The review included 52 studies, of which most focused on office or care workers. An increase in studies since 2020 indicates growing interest in the topic. Challenges among screen-level bureaucrats included work-life balance problems, technostress and fear of job loss. Among street-level bureaucrats, reported challenges included curtailed discretion, lack of user involvement and ethical stress. Identified knowledge gaps include the small number of studies with a work environment focus in general and on sectors beyond office and health settings in particular, few studies set outside of Europe and absence of studies on recent phenomena such as AI or algorithmic management.

**Conclusions:**

In view of the presented scarcity of research, we suggest that relevant questions are included in national and local surveys to enable more research, that more studies are conducted in occupational sectors, countries and regions lacking this type of research, and that comparative research is stimulated to uncover differences between the effects of digitalisation on occupational health in private and public sector work settings.

## Introduction

A growing literature on how digital technology affects the way we work shows that the same digital system or tool can have both negative and positive effects on the health of workers and their work environment, depending on how it is implemented.^1,2^ According to a literature review on digitalisation and psychosocial health, some of the negative effects are technostress, increased availability demands, work-private life interference and technology-related harassment.^1^

The public sector is interesting in this context because of the increasing pressure on national and local governments to digitalise services for the purpose of enhancing impartiality, transparency and efficiency.^3–5^ Another factor driving the digitalisation of public services is demographic change: ageing populations lead to increasing demands for services in societies where available workers are increasingly in short supply.^6^

There is a growing literature on how digitalisation affects government service quality,^7,8^ justice, and accountability,^9^ but few researchers have studied its effects on work, health and wellbeing of public employees.^10^

Departing from the assumption that safety and health of public employees are crucial components in delivering quality services and attracting and retaining staff, the objective of this article is therefore to present and discuss existing evidence and identify knowledge gaps related to how digitalization affects the work and work environment of public sector employees.

In this review, two definitions guided our work. The first, Digital technology, we define as different types of smart machines, robots and tools equipped with digital technology, for example software systems, animations and simulations.^2^ The second, Occupational health, we define as an area of work in public health to promote and maintain highest degree of physical, mental and social well-being of workers in all occupations.^11^

## Background

In academic and political discussions about digitalization in the public sector, e-Government (short for electronic government) often plays a central role. While the scope of this article includes e-Government, with its focus on information technology, the Internet, and computers, it also extends beyond those boundaries. In our endeavour to cover all aspects of how digitalization affects public sector employees, we also included more practical digital solutions used by front-line workers, i.e., workers in direct contact with customers or the general public, such as GPS, digital alarms, and digital keys. Despite some differences between e-Government and other digital solutions, the underlying rationale is the same: to enable public administrations to become more efficient in meeting citizen-centred service needs and responding to pressure from citizens and businesses.

Two concepts are used in this paper to categorise public sector employees: ‘street-level bureaucrats’, who interact closely with citizens,^12^ and ‘screen-level bureaucrats’,^13^ for whom information and communications technology (ICT) has partially or fully replaced face-to-face contacts. The theory of street-level bureaucrats^12^ is useful in this context because of its focus on the work of front-line employees, which is characterised by a balance between a degree of discretion to get work done and the necessity of operating within the rule of law. Standardisation of work tasks and handling of increasing amounts of sensitive data are just two of many aspects in digitalised work that can affect this balance.^14^ A pertinent question among street-level and screen-level bureaucrats alike is whether digitalisation curtails or enables frontline policy discretion.^14^

Health and wellbeing issues differ between these two groups. Musculoskeletal issues are for example common among screen-level bureaucrats due to computer work,^15^ while moral or ethical stress^16^ is more common among the street-level bureaucrats. Overall, work-related stress is a recurrent theme in both groups and in the literature on digitalisation and occupational health.^1^ The Job Demands-Resources theory,^17^ suggests that high demands caused by digital technology should be accompanied with enough resources to avoid employees are drained of energy and reduce their motivation, which could have consequences for employees’ health.^18^ Technostressors, described as techno-overload, techno-invasion, techno-complexity, techno-insecurity and techno-uncertainty,^19^ can be found in both groups. Digital discretion, when ICT fully or partly replaces discretion,^13^ is considered to increase efficiency and service quality by treating all citizens equally, thus potentially boosting employee motivation and wellbeing. But it can also lead to deprofessionalization^20^ and demotivation.^21^

Echoing Evangelia Demerouti,^18^ digitalization and automation can lead to healthy jobs if they are designed in a people-centric manner; people are in control of the technologies; and efforts are made to maximize job resources and keep job demands at manageable levels. Appropriate resources include managerial support, training, and sufficient number of staff,^17,19,22^ a focus on occupational health at all management levels of the organisations,^23,24^ a workplace culture that takes safety and health seriously^25^ and understanding that digital technology affects workers’ health and wellbeing.

## Methods

A scoping review is well suited for complex and multi-disciplinary topics such as this one.^26^ To ensure rigor, reproducibility, and transparency, we followed the methodological guidance for conducting a JBI scoping review.^26^ This guidance is aligned with the current standard for scoping reviews: the Preferred Reporting Items for Systematic Reviews and Meta-Analyses extension for Scoping Reviews (PRISMA-ScR).

### Eligibility criteria

Participants: All types of public sector employees were included as well as private sector employees who work for the public sector in rendering public services. This includes for example employees engaged in schools, healthcare, social services, and childcare.

Concept: All digital technology affecting the occupational health of public employees in any way were considered. We used the definitions of digital technology and occupational health presented earlier.

Context: Studies set in the public sector were considered as well as those in private companies contracted by local, regional, or national government.

Types of Sources: This scoping review considered all study designs including qualitative, quantitative, and mixed methodologies.

Review question: How do digital solutions affect occupational health of employees in the public sector?

Sub-questions: 1. Which digital solutions are being considered in the literature? 2. Which groups of employees/professions/sectors feature in the literature?; 3. In which countries and at what government level (national, regional, local) are the studies set?; 4. What positive and negative aspects of digitalisation on the occupational health of public employees feature in the literature?

### Review methods

**Search strategy**: Our search strategy aimed to locate studies published in peer-reviewed journals. Initial key words were developed in thorough discussions between the authors and four librarians at the Karlstad University Library. These words, for example e-government, local government and work environment, were tested in a limited search in EBSCO to identify articles on the topic. Text words contained in the titles and abstracts of relevant articles, and the index terms used to describe the articles, were subsequently used by the librarians to develop a full search strategy for PubMed, PsycINFO, Business Source Premier (EBSCO), and Scopus. The search strategies produced by the librarians (see Appendix 1) included the identified keywords and index terms and were adapted for each included database. We included studies published in English between 1 January 2010 and 31 December 2023.

**Figure 1.**
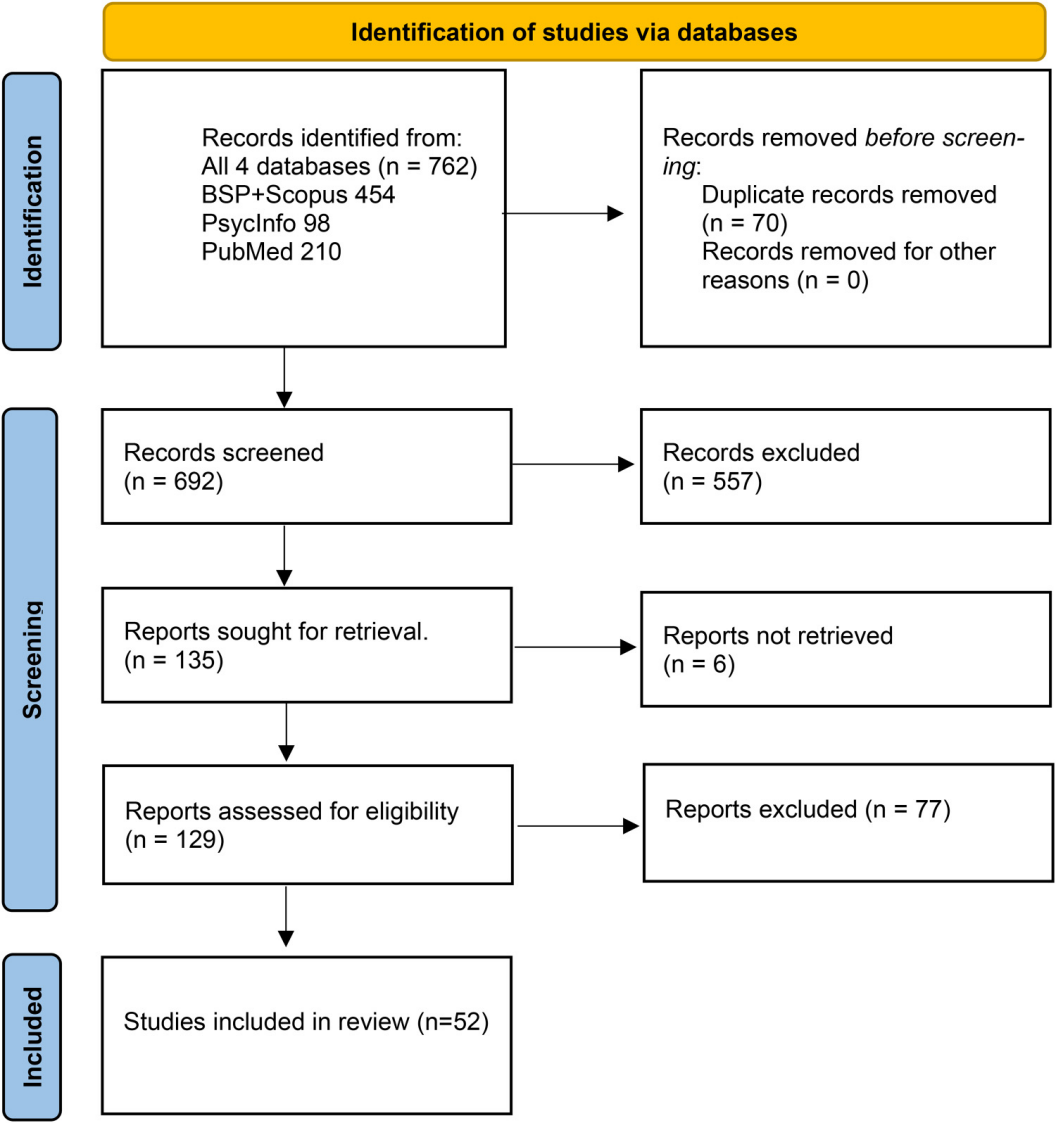
PRISMA 2020 flow diagram of the search and screening process of the scoping review.

**Evidence selection**: Following the search, all identified citations were collated and uploaded into the citation management system EndNote 20.3 (Clarivate Analytics, PA, USA) and duplicates removed. Following a pilot test, titles and abstracts were then screened by two (or three if there was a disagreement) independent reviewers (the authors) for assessment against the inclusion criteria for the review. To avoid bias, the pair constellations changed throughout the process. Potentially relevant sources were retrieved in full, and their citation details imported into the software programme Zotero. The full texts of the selected citations were assessed in detail against the inclusion criteria by two independent reviewers. Reasons for exclusion of sources of evidence at full text that did not meet the inclusion criteria were recorded and reported in the scoping review. Any disagreements that arose between the reviewers at each stage of the selection process were resolved through discussion between the three reviewers. The results of the search and the study inclusion process was reported in full in the final scoping review and presented in a Preferred Reporting Items for Systematic Reviews and Meta-analyses extension for scoping review (PRISMA-ScR) flow diagram. Figure 1 provides a PRISMA-ScR flow diagram of the search process.

**Data Extraction**: Data was extracted from papers included in the scoping review by two or three independent reviewers using a data extraction tool (tables in Microsoft excel) developed by the reviewers. The tool was piloted by each reviewer extracting data from the same ten publications and subsequently revised to delete characteristics considered irrelevant whilst adding others that the reviewers considered necessary. Data extracted included: full reference, country setting, government level (local/regional/national), studied population (profession/branch/role), methodology, technology featured in the study, and occupational health aspects included.

### Analysis and presentation

Out of the 692 identified titles and abstracts, 52 articles proceeded to data extraction (listed in Appendix 2). The evidence was presented in diagrams and tables, complemented by a narrative summary describing how the results relate to the review's objective and questions. Patterns emerging during the data extracting phase led the authors to divide the included studies in categories with an aim to improve the overview and understanding of the material. The first categorisation was between studies with 1) street-level or 2) screen-level bureaucrat focus. A subsequent categorisation was made by type of digital application that played the main role in the included studies: 1) organisational technology, for example management systems; 2) specific systems and tools, for example digital alarms; 3) mobility/ICT, for example smartphones.

## Results

Figure 1 illustrates the process that eventually resulted in 52 included articles.

This section starts by synthesising descriptive information of the included studies over time, types of workplaces, and digital technologies featured in them. It then proceeds to present the substance of the articles, including the study populations, digital technology discussed, and challenges and opportunities for occupational health appearing in the study results. In most cases the results are presented by dividing them in two groups: street-level (frontline workers in close contact with service recipients) and screen-level bureaucrats (back-office computer workers). This division allows us to present important differences in digital technology, challenges, and opportunities between the two groups.

### Descriptive aspects of the selected studies

**Figure 2.**
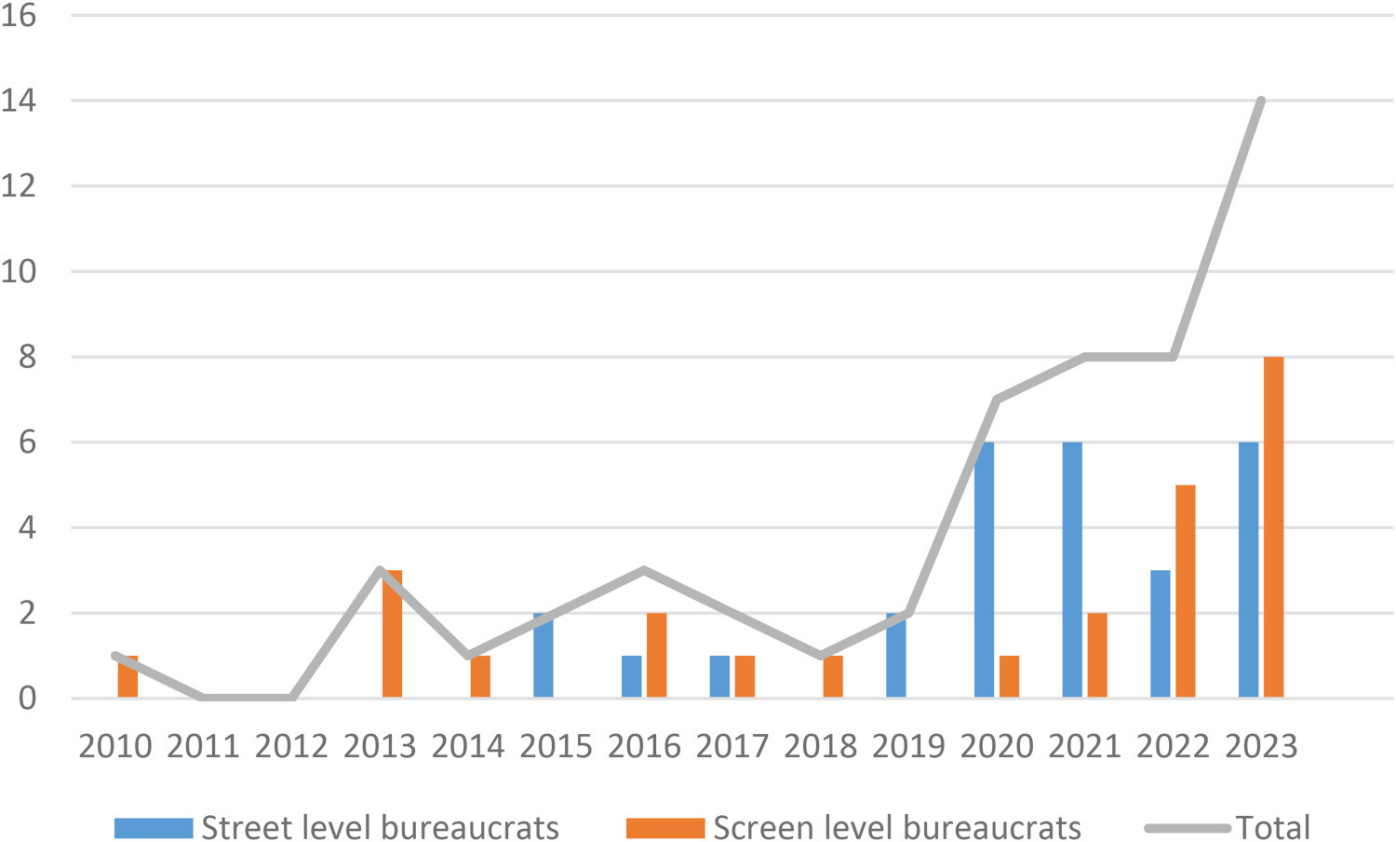
Included studies over time and distribution street- or screen-level bureaucrat focus (N).

The first aspect presented here are the number of included publications over time. This characteristic can be considered a proxy for researchers’, and perhaps also journals’ interest in studies on digitalisation and occupational health in the public sector. As indicated in Figure 2, the number of included articles was low during the years 2010–2019, with a significant increase starting in 2020.

**Figure 3.**
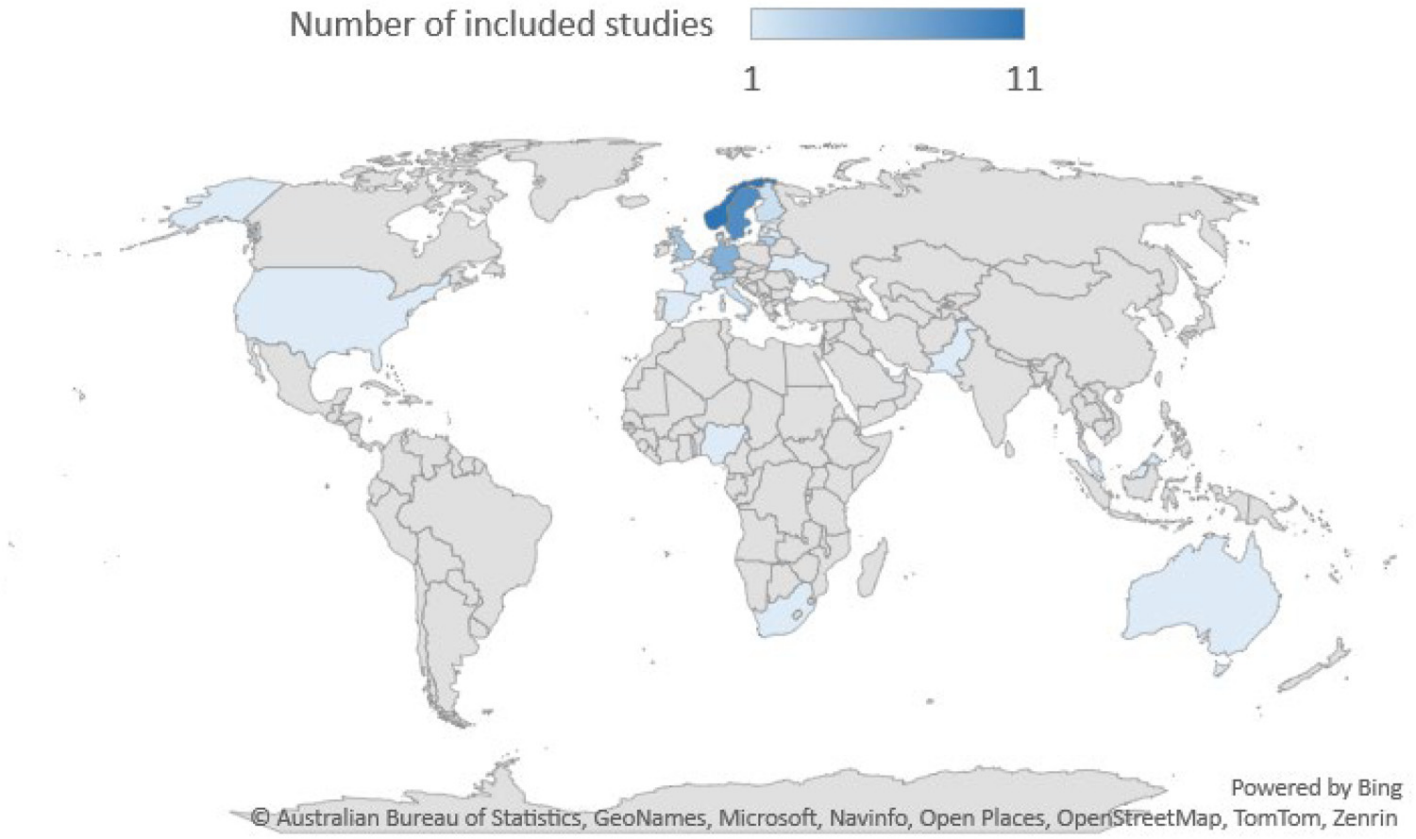
Country setting and number of included studies in each study (because some studies covered several countries, the total number exceeds the number of included articles).

The second aspect presented here is the geographical context of the studies. The geographical setting of the included 52 articles were performed in a broad range of countries (Table 1). As illustrated in Figure 3 below, most studies were set in European countries, with Scandinavia particularly well represented. Non-European studies were set in USA, Nigeria, South Africa, United Arab Emirates, Malaysia, Pakistan, and Australia.

**Table 1.** Characteristics of included studies: studies (N and %) total and divided by street- or screen-level bureaucrat focus.

Characteristics		Street-level bureaucrats	Screen-level bureaucrats	Total	%
*Geographical setting*	Americas	1		1	2
	Africa		2	2	4
	Asia/Oceania		4	4	8
	Europe	24	19	43	86
*Sector*	Care	18		18	34
	Office	2	25	27	52
	Other	6		6	14
*Research method*	Qualitative	21	10	31	60
	Quantitative	3	10	13	25
	Mixed	3	5	8	15
*Government level*	International	2	1	3	6,5
	National	4	7	11	23
	Regional	2	1	3	6,5
	Local	19	11	30	64

The third aspect presented here is the professional setting of the included articles (Table 1). Of the 27 studies focusing on street-level bureaucrats, all but six focused on health workers. The others examined labour inspectors, social workers, school staff and one-stop-shop staff (that is when several services are provided in one place, for example via a website or a mobile app). All the screen-level studies focused on office workers.

The fourth aspect presented here is the choice of methods in the included studies. There was a mixture of different approaches, with 60% using qualitative methods, 25% using quantitative methods, and 15% using mixed methods. A comparison between the street-level and screen-level studies shows that whereas use of qualitative methods dominated in the street-level studies (in 21 of the 27 studies), the distribution between qualitative, quantitative, and mixed methods was more evenly distributed in the screen-level studies.

A fifth aspect presented here is the level of government considered in the studies (Table 1). More than half of the studies (64%) were set at local government level. About a quarter of the studies (23%) were set at national level, and 3% were set at the sub-national government level.

### Content and results: effects of digital technology on work and the work environment

**Figure 4.**
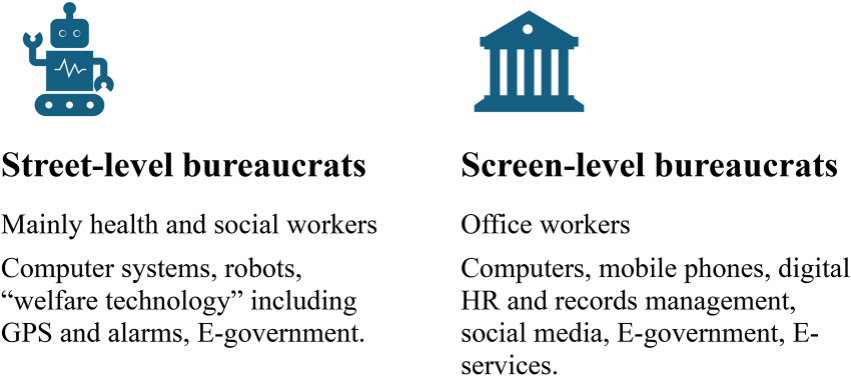
Summary of challenges and opportunities in the studies of digitalisation for the work and work environment of public sector employees.

This section presents the studied populations, technology, opportunities, and challenges in the included studies. Figure 4 provides a summary of positive and negative aspects of digitalisation appearing in the included studies.

In the following presentation the studies have been divided in different categories for increased overview. They are first divided in three categories based on the type of digital technology that receives most attention: 1) organisational; 2) systems and tools; 3) mobility and communication. Each category is then divided according to whether the studied employees can be described as having primarily: 1) street-level-, or 2) screen-level focus. To help the reader, a table summarising the findings has been added after the presentation of each of the three categories.

Among the 17 studies that focused on **organisational aspects,** eleven had street-level focus and six screen-level focus (see Table 2).

**Table 2.** Extracted information from included studies with focus on organisation, divided by street- or screen-level bureaucrat focus.

	Street-level studies (total n = 27)	Screen-level studies (total n = 25)
Organisation (n = 17)	(n = 11)	(n = 6)
*Population*	Healthcare professionals, counsellors, teachers, judges, tax officers.	Civil servants, office workers, HR professionals.
*Technology*	E-government, healthcare information systems, welfare technology, decision making technology.	E-services, E-government, HR management.
*Opportunities*	Improved information sharing, faster administration, improved quality of care, better overview.	Training improves usage of new systems.
*Challenges*	Increased work-load due curtailed discretion. Stress and ethical stress caused by poor design. Gender biased decision-making.	Poor work-life balance, conflicting demands, lack of knowledge, work complexity combined with low levels of autonomy.

*Populations* in focus in the street-level studies included four studies focusing on different types of healthcare personnel in home care or healthcare facilities.^27–31^ There was a mixture of study populations in two included literature reviews.^14,32^ The other articles examined on-line counsellors,^21^ teachers and school leaders,^33^ one-stop shop staff,^34^ and judges and tax administrators.^35^ *Populations* in the screen-level included office workers,^36–39^ case officers^40^ and HR managers.^41^

*Technology* featuring in the street-level studies included five studies with a focus on health services. One of those investigated the consequences of two badly integrated information systems.^27^ Four focused on how ICT used in welfare technologies (Concept used in Scandinavia to describe technology used to maintain or increase security, activity, participation or independence for people with a disability or the elderly.) impacts the attitudes and work environment of staff engaged mainly in elderly care.^28–31^ Some of the non-health studies focused on different aspects of E-government. Whereas two investigated the use of ICT more broadly to increase transparency, citizen participation, accountability, and efficiency,^14,21^ one focused more specifically on a one-stop-shop.^34^ Others were more occupation-specific, including one on the effects of digital tools used by teachers, causing red tape (excessive bureaucracy),^33^ one on on-line counsellors’ use of information systems,^21^ and one on judges and case workers in the tax administration using decision making technology in their work.^35^ Among the screen-level studies, one examined structure and flexibility related to digitalisation among white-collar workers.^36^ One looked at the effects of ICT and digital tools on the organisational climate, based on data from a large international survey.^39^ Three focused on the working conditions of public sector employees working with the provision of e-services.^37,38,40^

*Opportunities* with digitalisation for the work environment in the street-level studies included improved information sharing between hospital and municipal care services,^27^ faster administration,^28^ and improved quality of care and better overview of the organization.^30^ Some studies also described general satisfaction with the technologies.^29,31^ The screen-level studies showed how employees were more prone to use new systems if they had been included in the digitalisation process.^38^

*Challenges* with digitalisation in the street-level articles included curtailment of discretion,^14,35^ how frontline workers bend rules and work overtime to implement e-government at the expense of their wellbeing,^21^ how technology reduces work flexibility,^28^ decision-making imbalances due to assumption that men are more interested in technology and double work due to badly integrated systems,^27,28^ red tape from digital tools causing emotional exhaustion^34^ and ethical stress when technology is not adapted to the well-being of clients.^30^ The screen-level studies included challenges to the organisational climate,^39^ increased work complexity combined with low autonomy,^10^ frustration due to inadequate user expertise in how to handle the technology,^37,38^ and stress caused by poor work-life balance and conflicting work demands.^36^

The studies focusing on **systems and tools** (see Table 3), included 12 street-level and 10 screen-level studies (see Table 3).

**Table 3.** Extracted information from included studies with focus on systems and tools, divided by street- or screen-level bureaucrat focus.

	Street-level studies (total n = 27)	Screen-level studies (total n = 25)
Systems, tools (n = 22)	(n = 12)	(n = 10)
*Population*	Health and social care professionals.	Civil servants, IT experts, office workers, HR professionals.
*Technology*	Reminders and alarms, social and care robots, door locks, tools and patient recording systems to remotely monitor and manage treatment and care.	Chatbots, internet portal, digital HR and records management, computer work, enterprise resource planning systems.
*Opportunities*	Diminished workload, enhanced labour flexibility, enabled care in remote areas, good quality care.	Fewer routine tasks, efficiency, user satisfaction if employee involvement and self-efficacy.
*Challenges*	Curtailed discretion, ethical stress, low knowledge levels, poor system-server integration, alarms increasing workload and stress.	Fear of losing job and autonomy, pain, technostress, poor system-organisation fit, low user involvement.

*Populations* in focus in the street-level articles were, in all cases, health and social care professionals,^42–47^ including nurses and general practitioners,^48–51^ occupational and physical therapists,^49^ and elderly home care workers.^16,52^ *Populations* studied in the screen-level articles included public employees in general,^53–55^ IT and analytics staff,^56^ HR staff,^57–59^ case managers,^60^ and computer workers in general.^15,61^

*Technology* featuring in the street-level studies included digital tools and systems intended to facilitate the delivery of health and social care services as well as to improve the safety and quality of care services. Several studies focused on technology enabling care from a distance, such as the use of social alarms in nursing homes and in homecare^43^ and a palliative care application that enables staff to remotely monitor and manage patients’ safety, security, wellness, treatment, and care.^49^ One study explored similar use of technology in the elderly care during the Covid 19 pandemic, with a focus on videoconferencing tools, mobile phone messaging applications, and devices to entertain and stimulate older individuals to recall memories.^42^ Other studies focused on the impacts of digital safety alarm systems connected to GPS in users’ homes^44,48,51^ digital door locks,^48^ and digital medication dispensers in users’ homes.^16^ Among the studies in the context of institutional care delivery, one explored virtual reality, robotic assistive devices (designed to help people with disabilities or limitations in their daily activities), and social robots (capable of interacting with humans and other robots in long-term elderly care).^46^ Another study examined care robots for cognitive and social assistance in elderly care.^52^ One explored assistive technology for people with intellectual disabilities,^47^ and yet another studied electronic reminders in primary care.^50^ Technology featuring in the screen-level studies included AI assistants and the emergence of “algorithmic bureaucracy”, described as the transformation caused by algorithms of the socio-technical relationship between workers and their tools, and the organisation of this work.^56^ While two studies focused on the impact of digitalisation on HR management,^57,59^ others focused on electronic record systems,^54^ organizational internet portals,^55^ and resource planning systems.^53^ Two of the studies did not specify the types of tools or programmes but looked more generally at the impact of computer work on the health and work environment of the employees.^15,61^

*Opportunities* in the street-level studies included diminished workload and enhanced work flexibility,^42^ regained discretion as health professionals improved the script of a digital medication dispenser in the home care services,^16^ social alarms (alarm device installed in home of client) enabling remote care staff to feel closer to the patients^43^ and digital reminders among on-site staff, contributing to better care quality.^50^ The screen-level articles showed how digitalised HR practices freed caregivers from routine tasks,^56^ but required technological support and user involvement to result in improved satisfaction.^59^ Furthermore, technology self–efficacy (the belief in one's ability to succeed at something that involves the use of a technological tool) was seen as positive for intentions to use systems^57^ and as moderator in the associations between technostress and user satisfaction.^59^

*Challenges* in the street-level studies included moral and ethical challenges, such as clashes between demands to be efficient and innovative and to provide safe care^16^; how concerns about patient safety sometimes resulted in dissatisfaction and disempowerment^44,51^; how technical problems could have negatively effects on information-sharing^49^; how badly integrated systems and servers could cause stress^42,49^; and how beeping alarms could disturb nurses and patients^43^ and increase the workload.^50^ There were also organisational issues such as inadequate management attention to user involvement^48^ and training, which caused misunderstandings, lack of coordination^42,49^ and negative effects on workers’ competence.^48^ *Challenges* among the screen-level studies included fear of job-loss job due to digitalised HR practices^58^ and of reduced autonomy.^54^ Three studies focused on the associations between psychosocial and other work-related factors and musculoskeletal pain in computer work.^15,55,61^ Others described stress from systems that were not well adapted to the organisation of work^53^; pressure to use and adapt to new systems^55,59^; and insufficient resources in smaller municipalities, reducing their ability to involve users in technology design, thus risking higher administrative burden.^60^

Studies on **mobility and ICT** included four with street-level focus and nine with screen-level focus (see Table 4).

**Table 4.** Extracted information from included studies with focus on mobility and ICT, divided by street- or screen-level bureaucrat focus.

	Street-level studies (total n = 27)	Screen-level studies (total n = 25)
Mobility, ICT (n = 13)	(n = 4)	(n = 9)
*Population*	Labour inspectors, social workers, home care professionals.	Office workers including ministry officials, knowledge and administrative professionals.
*Technology*	Mobile technology, digital communication.	Social media, ICT-enabled mobile work.
*Opportunities*	More flexible and efficient work processes, increased work/life flexibility.	Improved organisational flexibility and resilience, socialisation and tacit knowledge sharing.
*Challenges*	Social isolation, curtailed discretion, strengthened power relations, non-reflexion, deskilling.	Stress from social media use, work-life balance challenges, lack of connectivity.

*Populations* in focus in the street-level studies included home care professionals,^62^ social workers^1,63^ and labour inspectors.^64^ *Populations* examined in two of the screen-level studies were administrative staff and knowledge professionals.^65,66^ A third study focused on administrative and technical staff.^67^

*Technologies* in the street-level studies included mobile technology rendering flexibility in space and time^62–64^ and communication via digital devices.^68^ The screen-level studies included various aspects of ICT-enabled mobile work,^65–67,69–71^ e-leadership,^72^ self-organization and digital leadership.^73^

*Opportunities* related to digitalisation reported in the street-level studies included increased efficiency and flexibility in time and space.^63,64,68^ Opportunities reported in the screen-level studies included organisational flexibility and improved response and resilience to the pandemic.^69^ One reported how mobile technology contributed to improved socialization and tacit knowledge sharing.^71^

*Challenges* in the street-level studies included social isolation and negative effects on learning^64^; communication challenges between practitioners and clients^63^; less reflection in favour of automated, non-critical reflexes due to reduced case complexity and curtailed spaces for discretion^64,68^; and the strengthening of existing power arrangements.^62^ Challenges in the screen-level studies included disappeared work-life boundaries in the nomadic practices of knowledge professionals^65^ and how workers with a family experienced more fragmented working time during the pandemic, while those without a family experienced longer working time.^66^ While one study discussed problems associated with being less connected than other workers at the workplace,^70^ another discussed the right to disconnect.^69^ Two studies focused on needs related to E-leadership during the pandemic for training, work-life balance, and the integration of digitalisation in the traditional organisational culture.^69,72^

## Discussion

The objective of this scoping review was to present and discuss existing evidence and identify knowledge gaps related to how digitalization affects the work and work environment of public sector employees.

### Increasing number of publications geographically and over time

From our descriptive results, we can see an increase in number of publications over time and a geographical expansion from mainly European countries to other parts of the world. Both trends point to increasing interest in the digital work environment of public employees across the world.

Our results show a trajectory from just a few included articles per year 2010–2019, to a strong and incremental increase after that. This growth was more noticeable among the street-level studies, which went from none before 2015 to the same number or more than the screen-level studies after that. The Covid 19-pandemic in 2020–2021 is a recurrent theme in many of the more recent studies and a plausible explanation for some of the increase. The incremental increase of street-level studies after 2020 is likely a result of technologies featuring in the studies, mainly from care service work, are relatively more recent compared to the digital technologies featuring in the screen-level studies.

Geographically, the included studies from South Africa, Malaysia and Pakistan published in 2023 broke the trend of studies set in high-income, predominantly European, countries. Although these studies examine comparatively early stages of digitalisation, they are nevertheless an indication of rising interest in the importance of employee health perspectives of digitalisation.

### Technology used by public sector employees

**Figure 5.**
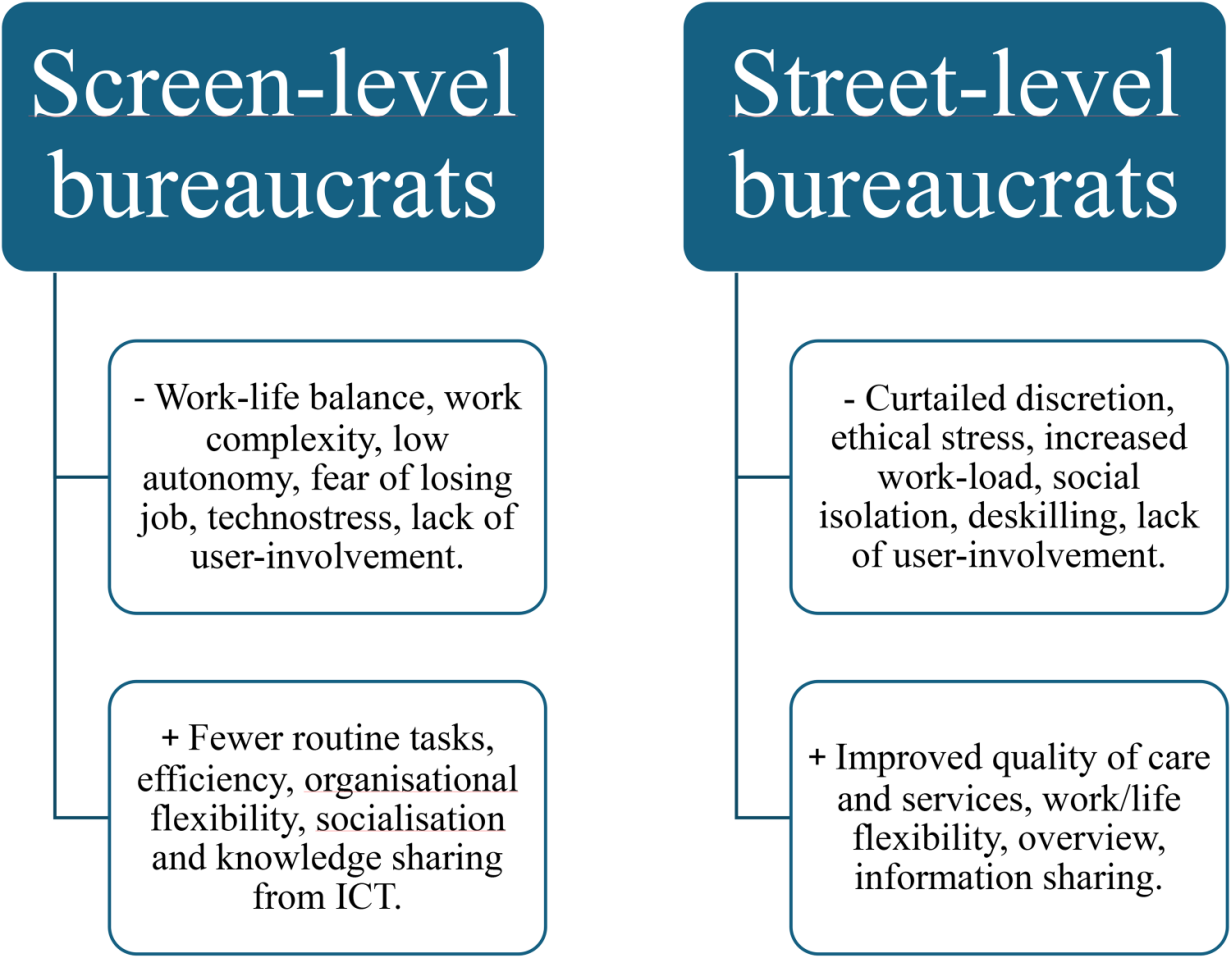
Main types of technology featured in the included studies divided by street-level or screen-level bureaucrat focus.

As for the content of the studies (illustrated in Figure 5), the types of digital technology figuring in the included studies were partly the same (computer systems and e-government) and partly different. One of the differences was that the screen-level studies examined changes in technology that has been around for decades, such as HR-systems gradually moving from digitalisation to automation and the introduction of AI. Most of the street-level studies, on the other hand, considered the more recent introduction of innovations intended to make the work of front-line public servants safer and more efficient. This trend is likely a consequence of increased use of digital technologies in all organizations, but probably also the result of the increasing pressure on national and local government organizations to digitalise services for the purpose of enhancing impartiality, transparency and efficiency.^3–5^ Another difference was a greater variety of digital tools among the street-level bureaucrats, for example digital locks and alarms, than among the screen-level bureaucrats, for whom digitalisation of work seemed limited to the computer and smartphone.

Because of the presented diversity of digital tools and systems in different types of work and workplaces it is difficult to draw general conclusions about how digital technology affects the work of public employees. This complex reality is further complicated by the fact that the same digital system or tool can have both negative and positive effects on the health of workers and their work environment, depending on how it is implemented.^1,2^ One likely practical consequence of this heterogeneity is that it complicates the work of employers, governments, and standard setting organisations in ensuring healthy and safe work environments.

### Effects of digitalization on the work and the work environment of public sector employees

Although many of the 692 titles and abstracts resulting from the search discussed digitalisation in the public sector, 52 had the required focus on the employees.

Studies in both groups observed that employees perceived digitalization as something positive that led to increased efficiency and quality of public service delivery, while also providing more job resources^17^ in the form of enhanced work flexibility and work-life balance. Reported downsides in the studies are in line with earlier research that shows how digitalization can lead to increased job demands,^17^ such as the intensification of work resulting in technostress for both groups (cf. 16).

Another example of reported downsides was ethical stress (cf. 15) among street-level bureaucrats due to the feeling of not having enough time to assist the clients. Not being able to perform your work duties due to time pressure is also in line with previous research on digitalization causing a curtailment of street-level bureaucrats’ discretion.^14^

A recurrent theme in the studies was work complexity, which can lead to increased workload and job demands.^17^ However, this complexity did not look the same. In the screen-level studies, automation and AI reportedly caused work tasks to disappear, resulting in a larger share of complex tasks and fear among the employees of job loss. In the street-level studies, focus was more on how increased work complexity led to increased workload – without concerns of job loss. Seen in a labour market perspective, it is reasonable to assume that these differences reflect the predicted reduction in simple office jobs and growing demand for labour in the care sector.

Linked to the standardization of work caused by digital systems and tools, several screen-level studies addressed problems related to reduced autonomy, which aligns with previous research^1^ and can be compared with the perceived curtailment of the discretion^13^ reported in several street-level studies. Reduced autonomy and curtailed discretion are central in the discussion of the included studies, as digital discretion could potentially boost employee motivation and wellbeing^14^ or, if it is curtailed, lead to demotivation^21^ and deprofessionalization.^20^

Articles in both groups also reported perceived social isolation, but in different forms. While work-life balance problems among office workers reportedly led to less social interaction with people outside of the workplace, street-level studies reported experiences of social isolation due to fewer colleagues or that physical presence at the workplace became redundant, affecting collegial socialisation.

The results from this review thus indicate that different types of technology in different public sector professions and workplaces, result in similar or different types of issues for the work and work environment of the employees, and this shows how important it is to continue this type of research. We suggest that more and better studies would help employers and policy makers take more considerate decisions when introducing digital solutions. The included studies highlighted recurrent issues related to a lack of knowledge and poor user involvement at the workplace level. These findings align with previous research emphasizing the importance of providing job resources to employees, such as training, support, and involvement in planning and implementing new technology.^17^ These resources help employees cope with job demands, including increased complexity and work intensification.^17^ Furthermore, these insights are consistent with research advocating for a safety and health focus at all management levels within organizations.^23,24^ A workplace culture that prioritises occupational health is crucial, especially considering the impact of digital technology on workers’ well-being.^25^

### Knowledge gaps

Although some of the included studies discussed aspects of AI, they were very few and not mirroring the recent increase of AI and algorithmic management in many workplaces. We believe that this reflects a change of language rather than substance, as many included studies focused on technologies including algorithms and AI without mentioning them explicitly.

A more glaring knowledge gap is the fact that only 52 out of almost 700 identified publications examined work and work environment from the point of view of the workers. The incremental increase since 2020 could be a sign of change.

The geographical representation of the studies, with only seven studies outside of Europe, is also problematic but the publication of several non-European studies in 2023 could be an indication of change.

Another knowledge gap detected in this review is the lack of studies in sectors outside of the care and office contexts. Although these sectors are important in the sense of their importance to the economy and as employer to many workers, effects of digitalisation in other public sector professions and work contexts also deserve to be examined. In this review, only six of the included studies examined “other” professions, including teachers, legal and tax officers, and labour inspectors.

### Study limitations

One methodological weakness is that the search strings focused on the public sector, thus including words like “government” and “public” (see appendix 1). We later realised that we may have missed out on profession-specific articles that did not include such words, for example studies on teachers. The decision to only include publications in English is another limitation as we may have missed out on articles in other languages. To include grey literature may likewise have resulted in interesting national or international reports on the topic.

Another limitation that we found out late in the process was that public sector employees go under different names due to inter-country variety in use of terminology. The different size and role of the public sector in different countries is likely also a contributing factor to the outcome of this review as it could explain the over-representation of Scandinavian studies, where the share of public employees in the overall labour market is high. For future reproductions of this review, we suggest inserting occupation-specific search terms and a wider array of government employee terminology so as not to miss relevant studies.

## Conclusions

The objective of this scoping review was to present and discuss existing evidence and identify knowledge gaps related to how digitalization affects the work and work environment of public sector employees. We have shown that despite a large interest in the literature related to the effects of digitalisation on public sector employees, comparatively few of these studies focus on the work and work environment of the employees. An incremental increase of this type of studies since 2020 could indicate a growing interest in these questions. However, we found that most studies were set in Europe, especially the Scandinavian countries, and focused on office or care workers. In view of national differences in prevailing financial, political and cultural preconditions of public services, it is difficult to draw general conclusions from national studies. Consequently, national and local level policy makers would benefit from more studies of this type that are carried out in settings outside of Europe and, in particular, Scandinavia. On a similar note, considering the different types and uses of technology as well as effects on work and the work environment in health and office settings presented here, we would like to suggest that future research also focuses on other professions and work settings, for example ambulating work beyond care services. In addition to the suggested widening of geographical and sectoral scope of future studies, we would also like to suggest that questions on the effects of digital technology on the work environment are included in workplace, national and international surveys, to facilitate future research. Another suggestion is to explore the specific challenges and advantages of public sector digitalisation by for example comparative case studies including public and private sector organisations carrying out similar services.

## Supplemental Material

sj-docx-1-wor-10.1177_10519815251320274 - Supplemental material for Effects of work-related digital technology on occupational health in the public sector: A scoping review Supplemental material, sj-docx-1-wor-10.1177_10519815251320274 for Effects of work-related digital technology on occupational health in the public sector: A scoping review by Carin Håkansta, Annica Asp and Kristina Palm in WORK
