# Supplementary material for: Effects of work-related digital technology on occupational health in the public sector: A scoping review
Source: Work. 2025 Mar 18;81(2):2477–90. doi: 10.1177/10519815251320274 (PMC12231812; doi:10.1177/10519815251320274)
Supplement: sj-docx-1-wor-10.1177_10519815251320274 - Supplemental material for Effects of work-related digital technology on occupational health in the public sector: A scoping review [file sj-docx-1-wor-10.1177_10519815251320274.docx]

**Appendix 1. Search strings**

PsycInfo

Block 1

e-government OR "online government" OR "digital government" OR ict OR "information and communication technology" OR "information and communications technologies" OR "information and communication technologies" OR "digital technology" OR "digital technologies" OR automatisation OR "information technology" OR "information technologies" OR "technological change" OR "technology changes" OR "welfare technology" OR "welfare technologies" OR "digital transformation" OR "communication technology" OR "communications technologies" OR "communication technologies" OR "technology, information"

Block 2

"local government" OR "local governments" OR "city government" OR "city governments" OR municipal* OR "public sector" OR "local authority" OR "local authorities" OR "governing authority" OR "governing authorities" OR "county government" OR "metropolitan government"

Block 3

"work environment" OR "work environments" OR "working environment" OR "working environments" OR "working condition" OR "working conditions" OR "work condition" OR "work condition" OR "occupational safety" OR "occupational health" OR "employee attitude" OR "employee attitudes" OR "job satisfaction" OR employee OR employees OR worker OR workers OR personnel OR "occupational groups" OR "occupational group" OR "workplace conditions" OR "workplace condition" OR "workplace environment" OR "workplace environments" OR "industrial health"

Pubmed

Block 1

e-government OR "online government" OR "digital government" OR ict OR "information and communication technology" OR "information and communications technologies" OR "information and communication technologies" OR "digital technology" OR "digital technologies" OR automatisation OR "information technology" OR "information technologies" OR "technological change" OR "technology changes" OR "welfare technology" OR "welfare technologies" OR "digital transformation" OR "communication technology" OR "communications technologies" OR "communication technologies" OR "technology, information"

Block 2

"local government" OR "local governments" OR "city government" OR "city governments" OR municipal OR "public sector" OR "local authority" OR "local authorities" OR "governing authority" OR "governing authorities" OR "county government" OR "metropolitan government"

Block 3

"work environment" OR "work environments" OR "working environment" OR "working environments" OR "working condition" OR "working conditions" OR "work condition" OR "work condition" OR "occupational safety" OR "occupational health" OR "employee attitude" OR "employee attitudes" OR "job satisfaction" OR employee OR employees OR worker OR workers OR personnel OR "occupational groups" OR "occupational group" OR "workplace conditions" OR "workplace condition" OR "workplace environment" OR "workplace environments" OR "industrial health"

BSP

“e-government” OR “online government” OR “digital government” OR ict OR “information and communication technology” OR “information and communications technologies” OR “information and communication technologies” OR “digital technology” OR “digital technologies” OR automatisation OR “information technology” OR “information technologies” OR “technological change” OR “technology changes” OR “welfare technology” OR “welfare technologies” OR “digital transformation”

AND

“local government” OR “local governments” OR “city government” OR “city governments” OR municipal* OR “public sector” OR “local authority” OR “local authorities” OR “governing authority” OR “governing authorities”

AND

“work environment” OR “work environments” OR “working environment” OR “working environments” OR “working condition” OR “working conditions” OR “work condition” OR “work condition” OR “occupational safety” OR “occupational health” OR “employee attitude” OR “employee attitudes” OR “job satisfaction” OR employee* OR worker*

Scopus

TITLE-ABS-KEY ( {e-government} OR {online government} OR {digital government} OR ict OR {information and communication technology} OR {information and communications technologies} OR {information and communication technologies} OR {digital technology} OR {digital technologies} OR automatisation OR {information technology} OR {information technologies} OR {technological change} OR {technology changes} OR {welfare technology} OR {welfare technologies} OR {digital transformation} )

AND

TITLE-ABS-KEY ({local government} OR {local governments} OR {city government} OR {city governmentts} OR municipal* OR {public sector} OR {local authority} OR {local authorities} OR {governing authority} OR {governing authorities} )

AND

TITLE-ABS-KEY ({work environment} OR {work environments} OR {working environment} OR {working environments} OR {working condition} OR {working conditions} OR {work condition} OR {work condition} OR {occupational safety} OR {occupational health} OR {employee attitude} OR {employee attitudes} OR {job satisfaction} OR employee* OR worker*)

**Appendix 2. The included 52 publications**

ADEBAYO, F. & BILQUIS, M. 2018. E-Governance and Effective Bureaucratic Practices in Nigeria Civil Service. *International Journal of Business & Administrative Studies,* 4**,** 105-121.

AMOAKO, R., JIANG, Y., ADU-YEBOAH, S. S., FREMPONG, M. F. & TETTEH, S. 2023. Factors influencing electronic human resource management implementation in public organisations in an emerging economy: An empirical study. *South African Journal of Business Management,* 54.

BAJRALIU, A. & QORRAJ, G. 2023. Digital Transformations’ Impact on Sustainable HR Management: Comparative Study of Work-Life Balance and Skill Development in Public Versus Private Sectors of a Developing Country. *Public Policy and Administration,* 22**,** 358-369.

BATT-RAWDEN, K. B., BJÖRK, E. & WAALER, D. 2017. Human factors in the implementation and adoption of innovations in health care services. A longitudinal case study on the introduction of new technology. *Innovation Journal,* 22**,** 2-25.

BATT-RAWDEN, K. B., BJØRK, E., WAALER, D. & BATT-RAWDEN, V. H. 2021. A qualitative study of user experiences from the implementation of new technology in healthcare services, Norway. *Innovation Journal,* 26.

BAUDIN, K., GUSTAFSSON, C. & FRENNERT, S. 2020. Views of Swedish elder care personnel on ongoing digital transformation: Cross-sectional study. *Journal of Medical Internet Research,* 22**,** 10.

BUFFAT, A. 2015. Street-Level Bureaucracy and E-Government. *Public Management Review,* 17**,** 149-161.

BUSCH, P. A. 2020. Crafting or mass-producing decisions: Technology as professional or managerial imperative in public policy implementation. *Information Polity: The International Journal of Government & Democracy in the Information Age,* 25**,** 111-128.

CECIL, E., DEWA, L. H., MA, R., MAJEED, A. & AYLIN, P. 2021. General practitioner and nurse practitioner attitudes towards electronic reminders in primary care: a qualitative analysis. *BMJ Open,* 11**,** e045050.

CHRISTENSEN, M., FREDERIKSEN, A. B. & MADSEN, K. B. 2022. What kind of social work in what kind of virtual space? *Nordic Social Work Research,* 12**,** 339-349.

CLAASSEN, K., RODIL DOS ANJOS, D., KETTSCHAU, J. P., WREDE, S. J. S. & BRODING, H. C. 2023. DigiFuehr 2.0: Novel insights for digital leadership. *Journal of occupational health,* 65**,** e12383.

CLOUET, H. 2022. Overtime or fragmentation? Family transactions and working time during the COVID-19 pandemic. *International Labour Review,* 161**,** 219-243.

DAVID, A., YIGITCANLAR, T., LI, R. Y. M., CORCHADO, J. M., CHEONG, P. H., MOSSBERGER, K. & MEHMOOD, R. 2023. Understanding Local Government Digital Technology Adoption Strategies: A PRISMA Review. *Sustainability (Switzerland),* 15.

ELFERING, A., DUBI, M. & SEMMER, N. K. 2010. Participation during major technological change and low back pain. *Ind Health,* 48**,** 370-5.

FRENNERT, S. 2019. Lost in digitalization? Municipality employment of welfare technologies. *Disability and Rehabilitation: Assistive Technology,* 14**,** 635-642.

FRENNERT, S. & BAUDIN, K. 2021. The concept of welfare technology in Swedish municipal eldercare. *Disabil Rehabil,* 43**,** 1220-1227.

GIRITLI NYGREN, K., AXELSSON, K. & MELIN, U. 2013. Public e‐services from inside: A case study on technology's influence on work conditions in a government agency. *International Journal of Public Sector Management,* 26**,** 455-468.

GLOMSÅS, H. S., KNUTSEN, I. R., FOSSUM, M. & HALVORSEN, K. 2020. User involvement in the implementation of welfare technology in home care services: The experience of health professionals—A qualitative study. *Journal of Clinical Nursing,* 29**,** 4007-4019.

HASSAN, M. K. & MOUAKKET, S. 2016. ERP and organizational change: A case study examining the implementation of accounting modules. *International Journal of Organizational Analysis,* 24**,** 487-515.

HENRIKSEN HAGEN, D. 2023. The effect of digitalization on the daily use of and work with records in the Norwegian public sector. *Records Management Journal,* 33**,** 105-119.

HOTI, H., HOTI, A. H. & KURHASKU, E. 2021. Impact of information technology on the HR practices in the public sector: Evidence from the Republic of Kosovo. *European Journal of Sustainable Development,* 10**,** 724-735.

HUMPHRY, J. 2014. Officing: Mediating time and the professional self in the support of nomadic work. *Computer Supported Cooperative Work: CSCW: An International Journal,* 23**,** 185-204.

HÅKANSTA, C. 2022. Ambulating, digital and isolated: The case of Swedish labour inspectors. *New Technology, Work and Employment,* 37**,** 24-40.

IBRAHIM, H., MOHD ZIN, M. L., AMAN-ULLAH, A. & MOHD GHAZI, M. R. 2023. Impact of technostress and information technology support on HRIS user satisfaction: a moderation study through technology self-efficacy. *Kybernetes*.

JEYASINGHAM, D. 2020. Entanglements with offices, information systems, laptops and phones: How agile working is influencing social workers’ interactions with each other and with families. *Qualitative Social Work: Research and Practice,* 19**,** 337-358.

JÄMSEN, R., SIVUNEN, A. & BLOMQVIST, K. 2022. Employees' perceptions of relational communication in full-time remote work in the public sector. *Comput Human Behav,* 132**,** 107240.

KALINIENE, G., USTINAVICIENE, R., SKEMIENE, L. & JANUSKEVICIUS, V. 2013. Associations between neck musculoskeletal complaints and work related factors among public service computer workers in Kaunas. *International Journal of Occupational Medicine and Environmental Health,* 26**,** 670-681.

KALINIENE, G., USTINAVICIENE, R., SKEMIENE, L., VAICIULIS, V. & VASILAVICIUS, P. 2016. Associations between musculoskeletal pain and work-related factors among public service sector computer workers in Kaunas County, Lithuania. *BMC Musculoskeletal Disorders,* 17**,** 1-12.

KLEIVEN, H. H., LJUNGGREN, B. & SOLBJØR, M. 2020. Health professionals' experiences with the implementation of a digital medication dispenser in home care services- A qualitative study. *BMC Health Services Research,* 20.

KOLTSIDA, V. & JONASSON, L. L. 2021. Registered nurses' experiences of information technology use in home health care - from a sustainable development perspective. *BMC Nurs,* 20**,** 71.

KUHLMANN, S. & HEUBERGER, M. 2023. Digital transformation going local: implementation, impacts and constraints from a German perspective. *Public Money and Management,* 43**,** 147-155.

LEMKE, F., EHRHARDT, K. & POPELYSHYN, O. 2021. Support and resistance of public officials towards current eGovernment initiatives – A case study on Ukraine and Germany. *Unterstützung und Ablehnung von Angestellten des öffentlichen Sektors gegenüber aktuellen eGovernment-Initiativen ‒ eine vergleichende Fallstudie über die Ukraine und Deutschland.,* 14**,** 61-80.

LINDELL, E., POPOVA, I. & UHLIN, A. 2022. Digitalization of office work – an ideological dilemma of structure and flexibility. *Journal of Organizational Change Management,* 35**,** 103-114.

LOESCHNER, I. 2017. The technology mismatch paradox of mobile e-mail access: When changed norms of responsiveness meet technology undersupply. *Information Society,* 33**,** 133-146.

LYDAHL, D. 2023. Good care and adverse effects: Exploring the use of social alarms in care for older people in Sweden. *Health (London)***,** 13634593231185260.

MARTÍN-PALOMO, M. T., GONZÁLEZ-CALO, I., LUCCHETTI, G. & BADANTA, B. 2024. Experiences of health and social professionals using care technologies with older adults during the COVID-19 pandemic: A qualitative study. *Public Health Nurs,* 41**,** 101-111.

MEIER, R., BEN, E. R. & SCHUPPAN, T. 2013. ICT-enabled public sector organisational transformation: Factors constituting resistance to change. *Information Polity: The International Journal of Government & Democracy in the Information Age,* 18**,** 315-329.

MELKAS, H., HENNALA, L., PEKKARINEN, S. & KYRKI, V. 2020. Impacts of robot implementation on care personnel and clients in elderly-care institutions. *Int J Med Inform,* 134**,** 104041.

MUYLAERT, J., DECRAMER, A. & AUDENAERT, M. 2023. How Leader's Red Tape Interacts With Employees' Red Tape From the Lens of the Job Demands-Resources Model. *Review of Public Personnel Administration,* 43**,** 430-455.

NIELSEN, J. A., ANDERSEN, K. N. & DANZIGER, J. N. 2016. The power reinforcement framework revisited: mobile technology and management control in home care. *Information, Communication & Society,* 19**,** 160-177.

NYGREN, K. G., AXELSSON, K. & MELIN, U. 2013. Public e-services from inside A case study on technology's influence on work conditions in a government agency. 26**,** 455-468.

OELSCHLÄGEL, L., DIHLE, A., CHRISTENSEN, V. L., HEGGDAL, K., MOEN, A., ÖSTERLIND, J. & STEINDAL, S. A. 2021. Implementing welfare technology in palliative homecare for patients with cancer: a qualitative study of health-care professionals' experiences. *BMC Palliat Care,* 20**,** 146.

PALUMBO, R. 2022. Does digitizing involve desensitizing? Strategic insights into the side effects of workplace digitization. *Public Management Review,* 24**,** 975-1000.

RASOULI, O., KVAM, L., HUSBY, V. S., RØSTAD, M. & WITSØ, A. E. 2023. Understanding the possibilities and limitations of assistive technology in health and welfare services for people with intellectual disabilities, staff perspectives. *Disabil Rehabil Assist Technol,* 18**,** 989-997.

RYBNIKOVA, I., JUKNEVIČIENĖ, V., TOLEIKIENĖ, R., LEACH, N., ĀBOLIŅA, I., REINHOLDE, I. & SILLAMÄE, J. 2022. Digitalisation and e-leadership in local government before COVID-19: Results of an exploratory study. *Forum Scientiae Oeconomia,* 10**,** 173-191.

SIAL, M. A., PAUL, Z. I., RAFIQ, Z. & ABID, G. 2023. Does mobile technology shape employee socialization and enable tacit knowledge sharing in public sector organizations. *Journal of Open Innovation: Technology, Market, and Complexity,* 9.

SMARADOTTIR, B. F. & FENSLI, R. W. 2019. Evaluation of Technology Use in an Inter-Disciplinary Patient-Centered Health Care Team. *Stud Health Technol Inform,* 257**,** 388-392.

STOKKE, R., MELBY, L., ISAKSEN, J., OBSTFELDER, A. & ANDREASSEN, H. 2021. A qualitative study of what care workers do to provide patient safety at home through telecare. *BMC Health Services Research,* 21.

THUNBERG, S., JOHNSON, E. & ZIEMKE, T. 2023. Investigating healthcare workers' technostress when welfare technology is introduced in long-term care facilities. *Behaviour & Information Technology*.

TODISCO, L., TOMO, A., CANONICO, P. & MANGIA, G. 2023. The bright and dark side of smart working in the public sector: employees' experiences before and during COVID-19. *Management Decision,* 61**,** 85-102.

TUMMERS, L. & ROCCO, P. 2015. Serving Clients When the Server Crashes: How Frontline Workers Cope with E-Government Challenges. *Public Administration Review,* 75**,** 817-827.

VOGL, T. M., SEIDELIN, C., GANESH, B. & BRIGHT, J. 2020. Smart technology and the emergence of algorithmic bureaucracy: Artificial intelligence in UK local authorities. *Public Administration Review,* 80**,** 946-961.
